# Case report: Complete atrio-ventricular block successfully reversed in newly diagnosed primary cardiac B-cell lymphoma

**DOI:** 10.3389/fmed.2023.1119286

**Published:** 2023-03-15

**Authors:** Jiahui Mao, Yitong Xu, Mingfang Zhu, Luqun Wang, Yu Hou

**Affiliations:** ^1^Department of Hematology, Qilu Hospital of Shandong University, Jinan, China; ^2^Shandong Provincial Key Laboratory of Immunohematology, Qilu Hospital of Shandong University, Jinan, China

**Keywords:** primary cardiac lymphoma, B-cell lymphoma, atrioventricular block, anthracycline, case report

## Abstract

Primary cardiac tumors are extremely uncommon and primary cardiac lymphoma (PCL) is an even rarer subset. A definite diagnosis can be delayed, which increases the likelihood of a poor prognosis. We report a case involving a 64-year-old male who presented with dyspnea, palpitation, and third-degree atrioventricular block (AVB) secondary to primary cardiac B-cell lymphoma that was diagnosed *via* endomyocardial biopsy (EMB) and multimodality imaging. Chemotherapy was initiated using rituximab, cyclophosphamide, vindesine, and prednisone (R-COP) followed by implantation of an artificial capsule pacemaker. Third-degree AVB vanished, and the subsequent cycle of treatment was adjusted as R-CDOP (rituximab, cyclophosphamide, doxorubicin liposome, vindesine, and prednisone), with aspirin and rosavastatin to prevent ischemic events. So far, the patient had a good clinical course and normal electrocardiogram. This case underscores the importance of EMB in the diagnosis of heart neoplasms. It is worth noting that anthracycline is not contraindicated in PCL.

## Introduction

Primary cardiac lymphoma (PCL) is a type of lymphoma that exclusively involves the heart and pericardium (1). A retrospective study of a 22 autopsy series by Reynen revealed a prevalence of 0.02% for primary cardiac tumors (2). PCL is even more unusual, with an incidence ranging from 1 to 2% among primary cardiac tumors (3). Primary diffuse large B-cell lymphoma is the most common histological subtype, accounting for 58% of cardiac non-Hodgkin lymphomas (4). PCL responds well to chemotherapy, with 61% of patients having a remission period. Surgical treatment functions as palliative therapy that temporarily relieves cardiemphraxis and provides additional time for chemotherapy (5). Chemotherapy is used in most patients with PCL, with R-CHOP (rituximab, cyclophosphamide, doxorubicin, vincristine, and prednisone) the primary regimen used (6). Despite the development of diagnostic technologies and treatments, the prognosis of PCL remains poor, with a median survival duration of 12 months after initial diagnosis (7). Hence, clinicians need to maintain a high vigilance index and provide prompt treatment, which improve the prognosis of patients.

Here, we describe a case of primary cardiac B-cell lymphoma that was diagnosed *via* EMB and treated with R-CDOP post pacemaker implantation. This case provides a good reference for the rational use of anthracycline drugs in the treatment of cardiac lymphoma, when cardiac function allows.

## Case presentation

A 64-year-old male presented to our cardiology outpatient clinic with a 2 month history of chest tightness and shortness of breath without fever. Two months prior to admission, he had developed dyspnea with no apparent cause lasting several minutes, which relieved spontaneously. On August 21, 2022, he experienced dyspnea accompanied by generalized weakness, which lasted for half an hour and could not be relieved. There were no other concomitant symptoms, including dizziness, headache, nausea, or vomiting. He visited another hospital 1 week prior to admission, and an ambulatory electrocardiogram (ECG) revealed a third-degree AVB. Chest computed tomography (CT) revealed multiple enlarged lymph nodes in the mediastinum, pericardial effusion, and a soft tissue density shadow in the pericardium. Coronary angiography indicated that the patient had right coronary artery (RCA) plaque infiltration with 70% and 50% mid-segment stenosis in the left anterior descending artery (LAD). The patient was referred to our hospital for further examination and treatment.

On admission to our hospital, the patient’s blood pressure was 125/63 mmHg and the heart rate was 50 beats per minute. His chest sounded clear, heart sounds on auscultation were obtuse and arrhythmic, and no pathological murmurs were heard on auscultation of valves. Palpation indicated normal heart border; no enlargement of the liver, spleen, or lymph nodes; and no edema in the extremities.

Laboratory tests were as follows: high-sensitivity troponin I, 43.53 ng/L (normal range: < 17.5 ng/L); and N-terminal brain natriuretic peptide precursor, 2,522.00 pg/mL (reference value was 0–300 pg/mL). Liver, kidney, and thyroid function and autoimmune antibodies were within normal ranges.

**Figure 1:**
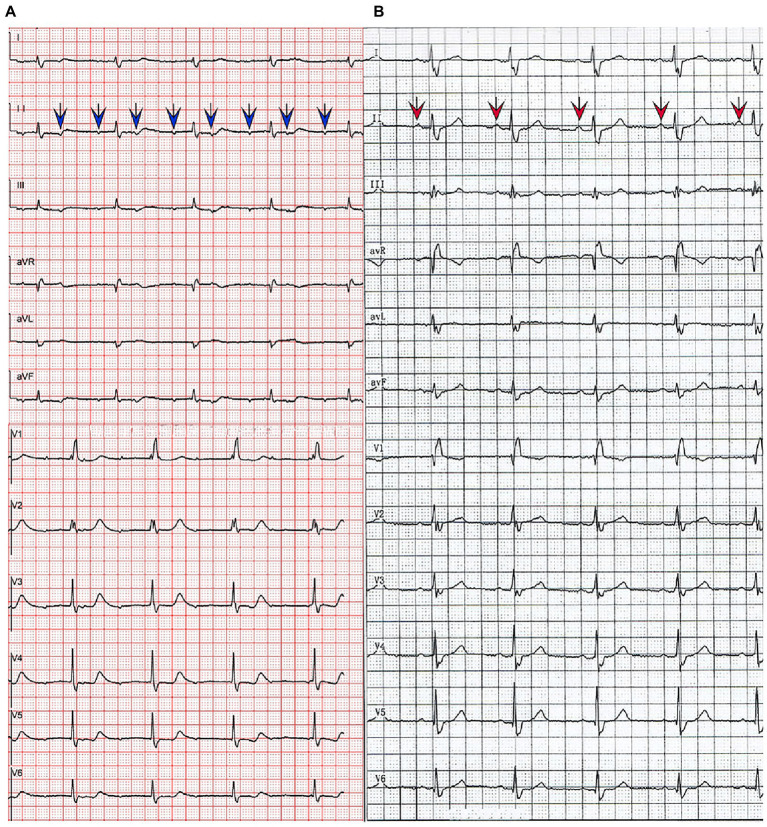
Electrocardiographic findings before **(A)** and after **(B)** treatment. The blue arrows indicate the patient’s blocked P-wave and the red arrows indicate the P-wave in sinus rhythm.

**Figure 2:**
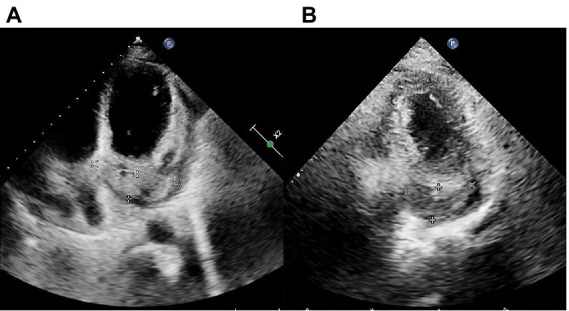
Transthoracic echocardiography (TTE) findings pre- and post-chemotherapy. **(A)** Pre-chemotherapy TTE revealing filling of the coronary sinus with inhomogeneous echogenic material measuring approximately 21.4 mm × 68.3 mm. **(B)** TTE after the second cycle of chemotherapy showing filling of the coronary sinus with inhomogeneous echogenic material measuring approximately 22.5 mm × 43.4 mm are shown.

**Figure 3:**
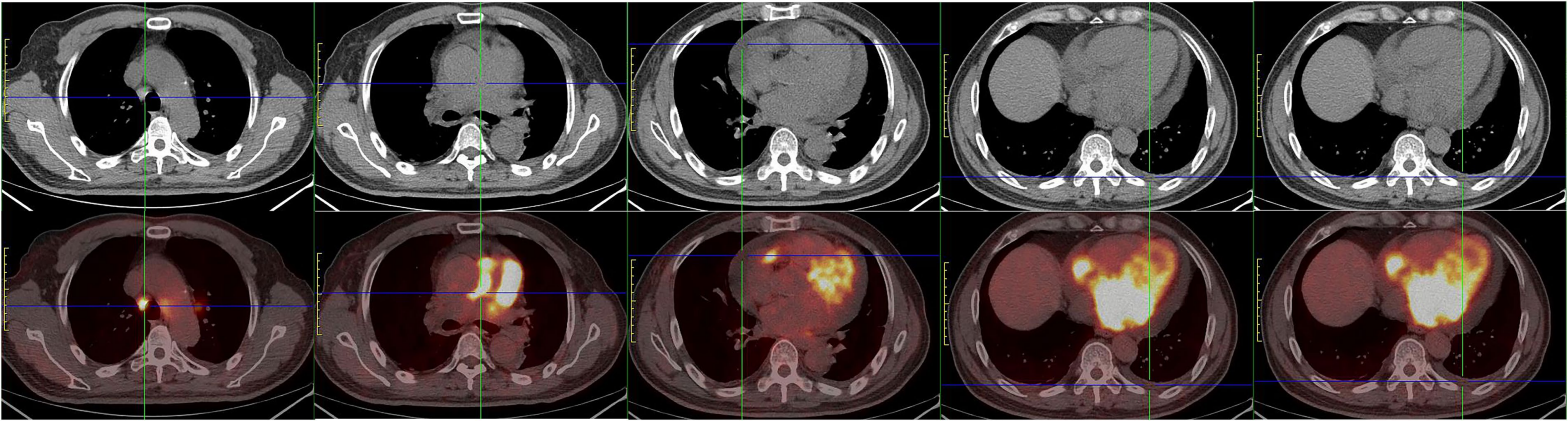
^18^F-fluorodeoxyglucose (FDG) positron emission tomography computed tomography (PET-CT) indicated multiple soft tissue nodules/masses with high FDG uptake in the pericardial cavity and a number of lymph nodes with high FDG uptake in the mediastinum.

The initial electrocardiogram (ECG) revealed a third-degree AVB, complete right bundle branch block, and junctional escape (Figure 1A). Transthoracic echocardiography (TTE) showed cardiac occupancy, left ventricular diastolic dysfunction, and pericardial effusion (Figure 2A). The patient was later admitted to the Department of Cardiology of our hospital with third-degree AVB and cardiac occupancy. ^18^F-fluorodeoxyglucose (FDG) positron emission tomography computed tomography (PET-CT) indicated the presence of multiple soft tissue nodules/masses in the pericardial cavity and high FDG uptake in the mediastinal lymph nodes, both suggesting a high probability of malignant lesions and possibly lymphoma (Figure 3). The patient was considered at high risk of tumors, so specialists from chemotherapy, hematology, and respiratory departments were consulted regarding the development of a biopsy protocol for the case. The patient was then transferred to the chemotherapy unit.

**Figure 4:**
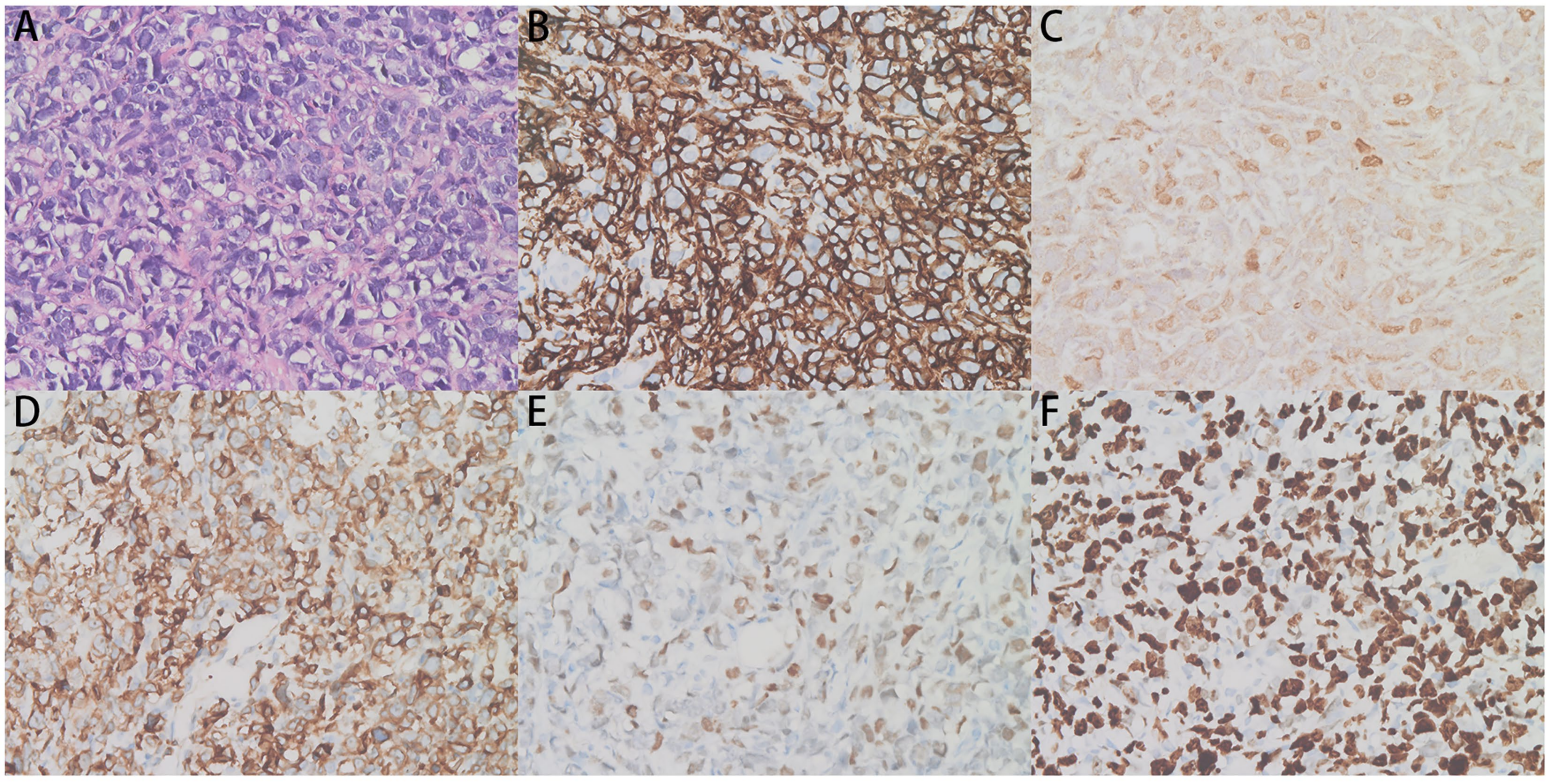
Pericardial biopsy showing irregular lymphocyte infiltration. **(A)** The hematoxylin and eosin (H&E)-stained tissues (×400) and **(B–F)** CD20 (+), c-Myc (2+, 40%), Bcl-2 (+), Bcl-6 (+), and Ki-67 (+) immunohistochemistry assays (×400) are shown.

After ruling out any contraindications, an EMB examination were performed, with ethical consent from the patient’s family. The patient’s pathological findings indicated (intrapericardial mass) diffuse large B-cell lymphoma (DLBCL) of a germinal center type. Immunohistochemical staining showed that the tumor cells were positive for CD20, CD99, Bcl-2, Bcl-6, LCA, INI-1, CD5 (scattered few cells +) and cell proliferation-related markers including Ki-67 (80%), MUM-1 (few cells +), and c-Myc (2+, 40%). However, tumor cells were negative for CD3, CD10, CD30, CD34, CD56, CD68, and CyclinD1. *In situ* hybridisation revealed that cells were Epstein–Barr virus encoded RNA negative (Figure 4). Given the patient’s clinical presentation, multi-modality imaging, histopathological and immunohistochemical analysis, the patient was diagnosed with primary cardiac B-cell lymphoma. The patient’s lesion was located in the pericardium and accompanied by mediastinal lymph node metastasis.

Considering the presence of third-degree AVB and the potential cardiotoxicity of anthracyclines, R-COP (rituximab, 600 mg on day 0; cyclophosphamide 1.3 g on day 1; vincristine, 5 mg on day 1; prednisone, 100 mg on days 1–5) was administered for the first cycle of chemotherapy. Two days later, the patient developed heart failure afterwards and his AVB still existed. Therefore, considering the opinion of the cardiology department and the patient’s own request, the patient subsequently underwent leadless pacemaker implantation (Micra) (Medtronic, Inc. Minneapolis, Minnesota, United States). As the patient’s cardiac symptoms faded and AVB disappeared (Figure 1B), a standard R-CDOP regimen was initiated (rituximab, 600 mg on day 0; cyclophosphamide, 1 g on day 1; doxorubicin liposomes, 20 mg on day 1; doxorubicin liposomes, 40 mg on day 2; vincristine, 4 mg on day 1; prednisone, 60 mg on days 1–5). Chest tightness and wheezing weakness were relieved after treatment, and echocardiography showed that the neoplasm on the pericardium also decreased in size (Figure 2B).

## Discussion

PCL is a rare primary malignant heart neoplasm that mainly affects the right chamber of the heart (1). The main clinical manifestations are pericardial effusion, heart failure, dyspnea, chest pain and rhythm disturbances (8). Complete AVB is the most commonly observed ECG change in patients with PCL (1). Among lymphomas with cardiac involvement, DLBCL is the most common histologic subtype (58%) and can be definitively diagnosed by histopathology (4, 9). When extracardiac evidence is lacking, PCL is easily mistaken for pericarditis, cardiomyopathy, rheumatic heart disease, coronary artery atherosclerotic heart disease, arrhythmia, or heart failure.

Achieving an accurate diagnosis at an early stage is important (10). Although imaging examinations are useful for detecting and characterizing masses in the heart, making a definitive diagnosis is difficult. Patient treatment and prognosis depend greatly on the tissue type and biological behavior of the tumor. Although fluid cytology of pericardial or pleural effusions is useful for rapid diagnosis, cardiac biopsy is considered when cytology is not possible (11, 12). EMB is a valuable tool for obtaining cardiac tissues (1, 13–15). It can be used for the diagnosis of intracardiac masses and arrhythmogenic cardiomyopathies (16). In our case, the patient’s ECG, TTE and PET-CT showed that the tumor was located in the pericardium. We performed an EMB on the patient and eventually confirmed the diagnosis of PCL. Early biopsy can speed up the initiation of effective treatment.

There is no consensus whether and when a pacemaker should be installed in PCL patients with AVB. Permanent pacemakers are sometimes unnecessary because AVB resolve in a proportion of PCL patients after immediate diagnosis and effective treatment (11, 17), especially in those without history of cardiovascular disease who restored normal sinus rhythm after chemotherapy (12, 18). However, there have been case reports of a temporary pacemaker being implanted to treat complete AVB (16, 17), and after chemotherapy the pacemaker was removed as the tumor vanished and the AVB disappeared (16). There was also a patient whose AVB did not subside after chemotherapy, and a permanent pacemaker was implanted (11). Our case provides another reference for the use of pacemakers in PCL patients with complete AVB.

Anthracyclines are cardiotoxic and may lead to left ventricular dysfunction or heart failure (19). Mandal et al. believed that drug-related cardiotoxicity may be reduced by omitting the anthracycline agent, doxorubicin (20). The first cycle of chemotherapy should be administered with caution because of the high risk of cardiac rupture during rapid tumor regression (18). Previous report suggested an initial R-COP regimen followed by sequential use of anthracyclines in PCL (21). Considering our patient’s cardiac condition, we adopted the R-COP regimen in the first cycle of chemotherapy. However, anthracycline is not always obliged to omit in PCL when cardiac function allows (12, 18), especially with Micra backup. The addition of an anthracycline was well tolerated in two PCL patients with AVB after pacemaker implantation (17, 22), which mirrors our case.

## Conclusion

In this case, we describe the clinical course of a patient diagnosed with primary cardiac B-cell lymphoma *via* EMB. Considering the cardiotoxicity of anthracyclines, we used R-COP instead of the traditional regimen during the first cycle of chemotherapy. Subsequently, an artificial pacemaker was successfully implanted, and third-degree AVB was resolved. After a comprehensive evaluation of the patient’s clinical condition, we added anthracycline to improve the chemotherapy effect. Currently, the patient’s cardiac function remains stable. This case provides a good reference for the rational use of anthracycline drugs in the treatment of cardiac lymphoma, when cardiac function allows.

## Data availability statement

The original contributions presented in the study are included in the article/supplementary material, further inquiries can be directed to the corresponding author.

## Ethics statement

The studies involving human participants were reviewed and approved by The Medical Ethics Committee of Qilu Hospital, Shandong University. The patients/participants provided their written informed consent to participate in this study.

## Author contributions

JM, YX, and MZ collected the data and drafted the manuscript. LW and YH edited the paper. YH supported the study and finalized the work. All authors contributed to the article and approved the submitted version.

## Funding

This work was supported by grants from National Natural Science Foundation of China (No. 81900121); Clinical Research Center of Shandong University (No. 2020SDUCRCC009); Graduate Education Reform Project of Shandong University (No. XYJG2020141).

## Conflict of interest

The authors declare that the research was conducted in the absence of any commercial or financial relationships that could be construed as a potential conflict of interest.

## Publisher’s note

All claims expressed in this article are solely those of the authors and do not necessarily represent those of their affiliated organizations, or those of the publisher, the editors and the reviewers. Any product that may be evaluated in this article, or claim that may be made by its manufacturer, is not guaranteed or endorsed by the publisher.
